# Young adult partner phubbing and relationship satisfaction: the mediating role of attachment anxiety and the moderating role of constructive conflict coping style

**DOI:** 10.3389/fpsyg.2025.1490363

**Published:** 2025-02-25

**Authors:** Yichu Han, Xin Li, WeiLing Song, Yifan He

**Affiliations:** ^1^School of Teachers Education, Huzhou University, Huzhou, China; ^2^School of Educational Science, Harbin Normal University, Harbin, China; ^3^School of Foreign Language, Heilongjiang College of Business and Technology, Harbin, China

**Keywords:** young adult partner, phubbing, relationship satisfaction, conflict coping style, attachment anxiety

## Abstract

**Introduction:**

This study investigates the association between young adult partner phubbing and relationship satisfaction, with a focus on the mediating role of attachment anxiety and the moderating role of constructive conflict coping styles (voice or loyalty). Understanding these dynamics is crucial for enhancing relationship satisfaction among young adults.

**Methods:**

A sample of 837 undergraduate students (376 male students; average age 21.02 ± 1.931 years) was recruited to complete questionnaires assessing young adult partner phubbing, relationship satisfaction, attachment anxiety, and constructive conflict coping styles. The data were analyzed to explore the mediating and moderating effects within the proposed model.

**Results:**

The findings revealed that young adult partner phubbing was negatively associated with relationship satisfaction. This relationship was mediated by attachment anxiety, indicating that higher levels of phubbing increased attachment anxiety, which in turn decreased relationship satisfaction. Additionally, the relationship between attachment anxiety and relationship satisfaction was moderated by constructive conflict coping styles, suggesting that individuals who employed voice or loyalty coping strategies experienced a less pronounced negative impact on their relationship satisfaction.

**Discussion:**

This study enhances our understanding of the mechanisms through which partner phubbing affects relationship satisfaction. The findings highlight the importance of addressing attachment anxiety and promoting constructive conflict coping strategies to mitigate the negative effects of phubbing. Practical implications for improving relationship satisfaction among young adult partners are discussed, emphasizing the need for interventions that foster healthy communication and conflict resolution skills.

## 1 Introduction

With the rapid development of information technology, mobile devices have become indispensable virtual organs for people, especially young people. Although the emergence of mobile phones has made our lives more convenient, when we become addicted to using them, it introduces hidden dangers. Phubbing is a relatively new term that stands for ‘phone snubbing,’ describing the act of snubbing someone in a social setting by looking at the phone instead of paying attention to them (Ugur and Koc, 2015). Phubbing is a common practice among young people (Vanden Abeele et al., 2019). Moreover, when this phenomenon occurs in young couples, it is called young adult partner phubbing. In fact, young adult partner phubbing occurs when both partners are physically present but one is mentally absent due to excessive mobile phone use. This is an interactive behavior, rather than a one-way behavior. Partner relationships perceived as ‘indifferent’ can be particularly distressing, with subsequent negative consequences for health (Ross et al., 2018).

In the context of the internet era, interpersonal communication has become a growing concern, both in virtual and real-life settings. The widespread use of smartphones and tablet PCs has significantly impacted interpersonal interactions, particularly among young couples. Research has shown that smartphones can diminish the quality of these interactions (Dwyer et al., 2018). Furthermore, mobile internet devices often lead to social isolation, reducing essential communication between individuals. Although smartphones can enhance the speed, quality, and effectiveness of communication, excessive reliance on these devices may lead to psychological barriers, such as phubbing. This behavior can create distance between friends and family members (Anshari et al., 2016). Given that young people are the primary users of smartphones, phubbing is particularly prevalent among this demographic. In fact, technological devices (e.g., computers, smartphones, or TVs) frequently interrupt interactions between partners, leading to increased conflicts, depressive symptoms, and lower life satisfaction (Mcdaniel and Coyne, 2016).

Young adult partner phubbing may exacerbate marital conflicts and anxiety. For instance, when one partner is physically present but mentally absent during communication, it can arouse suspicion and fear in the other, escalating neglect into conflict. In addition, the lack of necessary communication can block the communication process between couples, potentially leading to the breakdown of the relationship. Therefore, young adult partner phubbing has emerged as a significant negative trigger, seriously threatening the quality and satisfaction of relationships among young couples. These findings highlight the importance of addressing phubbing to prevent romantic relationship crises and promote sustainable relationships among young adults.

However, previous studies have primarily focused on the occurrence and harm of phubbing at the individual level (Karadağ et al., 2015). Some research has demonstrated that shared phone use can mitigate the adverse effects of phubbing through positive conflict resolution approaches (Beukeboom and Pollmann, 2021). Furthermore, the mobile phone exclusion effect caused by phubbing may threaten individuals’ basic psychological needs, particularly the sense of belonging (Chotpitayasunondh and Douglas, 2018). Thus, the present research aimed to examine the effect of young adult partner phubbing on relationship satisfaction, with a focus on the mediating role of attachment anxiety and the moderating role of constructive conflict coping styles.

## 2 Literature review

### 2.1 Partner phubbing and relationship satisfaction

Some previous studies have demonstrated that partner phubbing is negatively associated with relationship satisfaction (Beukeboom and Pollmann, 2021; David and Roberts, 2021). However, other findings have indicated that partner phubbing is not significantly related to relationship satisfaction (Cizmeci, 2017; Wang et al., 2021). Although there is no consensus among previous studies, the phubbing phenomenon has been shown to hinder personal relationships, potentially altering the fabric of social interactions (Błachnio et al., 2021). Intimacy develops through interactions in which one individual discloses personal information, thoughts, and feelings to a partner, receives a response, and interprets that response as understanding, validating, and caring (Laurenceau et al., 1998). Partner phubbing may hinder positive interactions between partners, leading to cracks in intimate relationships. As a result, interdependence theory offers a new perspective for understanding the relationship between partner phubbing and relationship satisfaction.

In fact, phubbing is essentially a subtype of social exclusion, stemming from the overuse of digital devices (e.g., tablets, mobile phones, and VR). The behavior of young adult partners is influenced by both themselves and their partners (Kelley et al., 2023). During communication, young adult partners often keep their heads down to use their mobile phones, leading to a lack of eye contact with their partners. This behavior can trigger negative feedback (Nakamura, 2015). Previous studies have found that phubbing is less harmful when it occurs during speaking than during listening. In addition, phubbing initiated in response to a notification is less harmful than proactively initiated phubbing (Vanden Abeele et al., 2016; Vanden Abeele and Postma-Nilsenova, 2018). Moreover, phubbing occurring more frequently is more harmful than phubbing occurring just once (Knausenbergerer et al., 2022). Empirical research has consistently shown that phubbing strongly contributes to feelings of ostracization (Knausenbergerer et al., 2022; McDaniel and Wesselmann, 2021). Recently, Vanden Abeele et al. (2024) tested expectancy violations theory as an explanation for the negative effects of phubbing. Their findings indicated that more intensive phubbing leads to lower perceptions of attentiveness, conversation quality, and relationship quality, although it does not significantly affect basic psychological needs (Chotpitayasunondh and Douglas, 2018; Knausenbergerer et al., 2022; Vanden Abeele et al., 2024). In other words, when partner phubbing occurs, the neglected partner experiences reduced intimacy, which, in turn, lowers relationship satisfaction (Halpern and Katz, 2017). Partner phubbing has a particularly strong impact on the quality of romantic relationships among young adult partners. This is because young adults are more adaptable to emerging technologies and tend to have more fragile relationships compared to older couples. Negative feedback resulting from partner phubbing is more likely to hinder young partners’ expectations of building positive relationships with one another. Therefore, expectancy violations theory may also provide a useful framework for explaining the feedback mechanism between partner phubbing and relationship satisfaction.

The relationship between partner phubbing and relationship satisfaction is also affected by self-esteem and marital status (Wang et al., 2021). Individuals with low self-esteem usually assume that their partners view them in the same negative way they view themselves (Murray et al., 2000). Whether partner phubbing affects relationship satisfaction depends on individual subjective feelings, including emotional responses triggered by negative feedback and personal evaluations of the situation. In addition, there is a marginally significant difference in the impact of partner phubbing between married and unmarried adults. This slight difference stems from the different cognitive mechanisms of younger and older partners. Young adult partners tend to be more sensitive to the emotional problems caused by phubbing (Miller-Ott and Kelly, 2015), whereas older partners are more concerned with the implications of phubbing behavior, such as disloyalty and unfairness (Clayton, 2014). Therefore, the mechanisms of phubbing and relationship satisfaction among young adult partners may require further exploration.

### 2.2 Attachment anxiety as a moderator

Romantic attachment mainly refers to the mutual support and emotional bond between partners, which fosters a sense of security and belonging (Lu et al., 2009). Specifically, adult attachment styles include attachment anxiety and avoidance, and these styles exhibit reliable individual differences among adults (Crowell et al., 2016). Among them, attachment anxiety is more closely related to relationship needs and dependence on intimacy, accompanied by a lower sense of self-worth and high levels of negative emotions (Shaver and Mikulincer, 2002). We focused on attachment anxiety because individuals with attachment anxiety tend to have lower relationship satisfaction with their partner (George et al., 2020).

Attachment anxiety is not solely a result of maladaptive romantic relationships, rather it is rooted in negative childhood experiences. In addition, intimate partner behaviors have been linked to attachment styles, particularly attachment anxiety (Sullivan et al., 2023). The results of a meta-analysis showed that higher marital quality is associated with better health status (Robles et al., 2014). This suggests that relationship satisfaction plays a significant predictive role in the physical and mental health development of adults, particularly among young adult partners. Another meta-analytic study indicated that attachment anxiety is negatively associated with relationship satisfaction (Candel and Turliuc, 2019). Adults with higher attachment anxiety scores tended to seek more positive feedback about their romantic relationships, yet they were also more likely to incorporate negative feedback into their self-views and experience stronger negative emotional reactions to such feedback (Carnelley et al., 2007; Dykas and Cassidy, 2011). It is reasonable to assume that phubbing, as a form of social exclusion, threatens individuals’ basic psychological needs and negatively impacts the quality of interpersonal relationships (Chotpitayasunondh and Douglas, 2018).

In addition, a meta-analysis by Zhang et al. (2022) found a strong positive association between attachment anxiety and mobile phone addiction, which was not moderated by gender. This suggests that the use of media devices, such as mobile phones, may activate and sustain individuals’ attachment anxiety. According to attachment theory, individuals develop working models or social expectations based on their developmental histories of seeking support, which influence how they perceive and react to close relationships (Shaver and Mikulincer, 2002). Media devices, including mobile phones, can provide emotional satisfaction and psychological support, acting as compensatory attachment objects. However, when one partner maintains closeness with others through smartphones (e.g., calls, text messages, and social media) while neglecting their current partner’s need for affection, it can exacerbate attachment anxiety (Sun and Miller, 2023). Therefore, we proposed that attachment anxiety mediates the impact of partner phubbing on relationship satisfaction.

### 2.3 Constructive conflict coping style as a mediator

Previous studies have preliminarily explored the relationship between phubbing and romantic relationship satisfaction; however, conflicting views remain regarding the nature and strength of this association. Moreover, the role of constructive conflict coping styles in mitigating the conflicts caused by phubbing among young adult partners remains unclear. Coping strategies refer to the internal resources individuals employ, involving emotional, cognitive, and behavioral efforts, to manage or mitigate stressors in specific situations (Lazarus and Folkman, 1984). A significant relationship was found between an individual experiencing phubbing and their partner’s perceived smartphone use conflict, regardless of gender (Denecker et al., 2004). Although previous studies have shown a significant correlation between conflict coping style and attachment anxiety, they have not consistently predicted marital quality (González-Ortega et al., 2017). However, for couples who used positive conflict coping styles, they had high marital quality (Li and Yang, 1990). During interactions between partners, if one provides vague or insufficient support, individuals with higher attachment anxiety are more likely to focus on negative information, leading to misunderstandings (Collins and Feeney, 2004). Those who feel ignored may perceive that their partner prioritizes their phone over them, which can further diminish relationship satisfaction. In addition, evidence suggests that mutual respect and love between spouses are key predictors of marital happiness (Danesh and Hydarian, 2006).

As discussed above, romantic relationships are influenced by uncertainty. According to the uncertainty reduction theory (Berger and Calabrese, 1974), feelings of uncertainty in romantic relationships may prompt individuals to use passive, active, and interactive strategies to gain information about their partner. Therefore, a constructive conflict coping style may reduce attachment anxiety and enhance relationship satisfaction and ultimately alleviate uncertainty in romantic relationships. Relevant studies have tested the moderating role of constructive conflict coping styles, finding that loyalty can moderate the relationship between attachment anxiety and relationship satisfaction (Mcdaniel and Coyne, 2016). However, the length of the romantic relationship does not moderate the relationship between social networking site use and disloyalty behavior (Clayton, 2014). Based on these findings, it is hypothesized that the mediating effect of attachment anxiety in the association between young adult partner phubbing and relationship satisfaction is moderated by constructive conflict coping styles (e.g., loyalty and voice). Furthermore, this mediating effect is expected to be stronger among individuals with high levels of constructive conflict coping styles.

## 3 The current study

**Figure 1:**
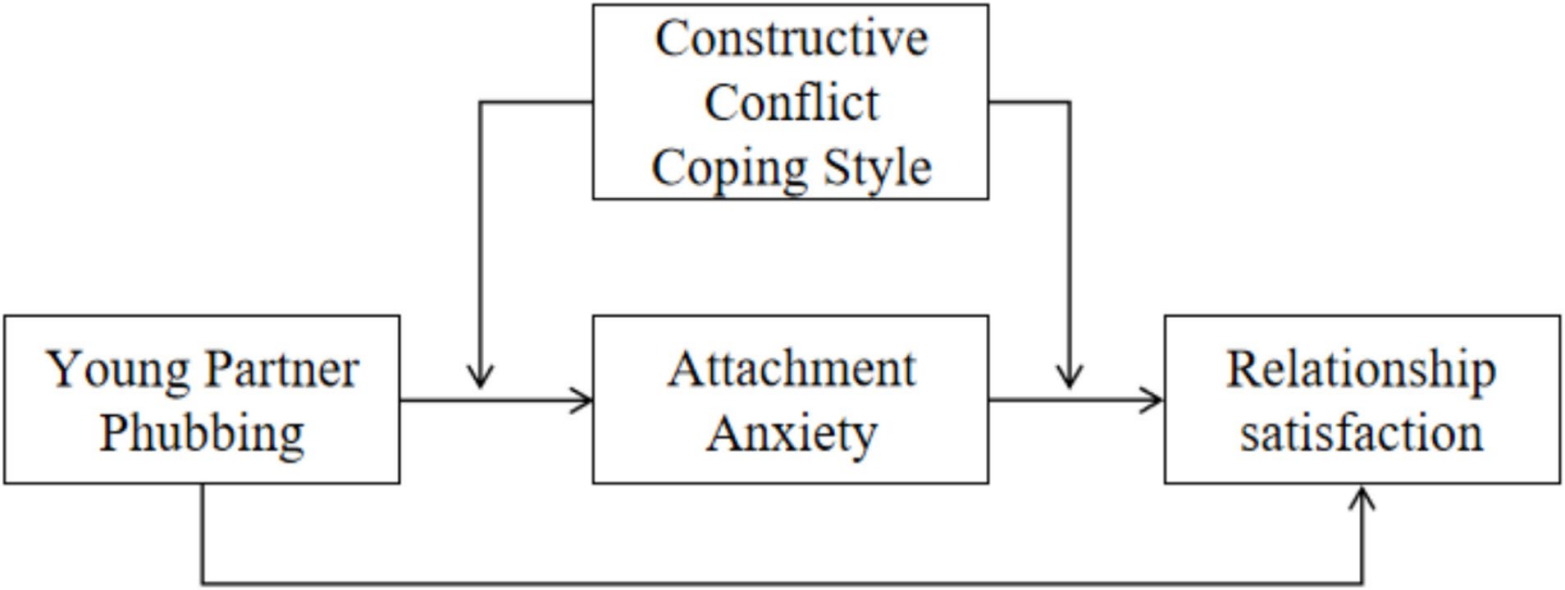
The conceptual model.

In conclusion, the present study aimed to examine the relationship between young adult partner phubbing and relationship satisfaction, as well as the mediating role of conflict coping styles and the moderating role of romantic partner attachment. These research questions mentioned in Figure 1. Form the basis of the conceptual model (see Figure 1).

Young adult partners, particularly those in the college student population, are a key demographic for studying the effects of partner phubbing on relationship satisfaction (Beukeboom and Pollmann, 2021). To explore the intrinsic associations between these variables, this research employed a questionnaire-based approach.

Based on the previous literature, the following hypotheses were proposed:

*H1*: Young adult partner phubbing is negatively related to relationship satisfaction.

*H2*: Partner attachment mediates the relationship between young adult partner phubbing and relationship satisfaction.

*H3*: Conflict coping styles moderate the mediating effects of partner attachment, with stronger effects observed among individuals with higher levels of constructive conflict coping styles.

## 4 Materials and methods

### 4.1 Participants

Convenience sampling was used in this study to recruit undergraduate students (*N* = 2052) through university social platforms (e.g., WeChat), libraries, classrooms, and other channels. Voluntary participants who were at least 18 years old and had a relationship of at least 6 months were recruited. After excluding individuals who did not meet the inclusion criteria or did not complete the questionnaire, the final sample consisted of 837 participants (*N* = 837). The majority of the sample included female participants (*n* = 461, 55.1%), compared to male participants (*n* = 376, 44.9%). The mean age of the participants was *M* = 21.02 years (SD = 1.931). The average duration of relationships in the sample was 23 months, and the average number of the relationships was 2.4.

### 4.2 Measurement

#### 4.2.1 Partner Phubbing

The Partner Phubbing Scale (Roberts and David, 2016), a widely used scale in recent years, was adopted to measure partner phubbing, using nine items (e.g., If there is a lull in our conversation, my partner will check his or her cell phone). The participants rated on a 5-point scale (1 = never; 2 = seldom; 3 = every now and then; 4 = often; and 5 = always) the extent to which a number of behaviors occurred, when they, in general, thought about moments with their partner. We translated the scale and generated the Chinese versions. It demonstrated good validity and reliability in the current study. The confirmatory factor analysis revealed an acceptable fit *χ^2^/df* = 4.117, RMSEA = 0.061, SRMR = 0.047, CFI = 0.967, TLI = 0.949, and Cronbach’s *α* = 0.746.

#### 4.2.2 Relationship satisfaction

The Relationship Assessment Scale (Hendrick, 1988) was adopted to measure the participants’ satisfaction with their romantic relationship, using seven items (e.g., to what extent has your relationship met your original expectations), including two reverse-scored items. The participants rated the items on a 5-point scale (1 = hardly at all; 2 = seldom; 3 = neither more nor less; 4 = more; and 5 = completely). The scale demonstrated good validity and reliability in the current study. The confirmatory factor analysis revealed an acceptable fit: *χ^2^/df* = 3.027, RMSEA = 0.049, SRMR = 0.024, CFI = 0.994, TLI = 0.99, and Cronbach’s *α* = 0.859.

#### 4.2.3 Attachment anxiety

The Chinese adaptation of the ECR Scale (Li and Kato, 2006) was widely used to measure romantic partner attachment with 36 items (e.g., I feel resentful when lovers do not spend time with me). The scale has two dimensions: attachment anxiety and attachment avoidance. The attachment anxiety scale was used to measure attachment anxiety with 18 items, each describing aspects of the romantic relationship. The participants rated each item on a 7-point scale, where 1 represented “strongly disagree” and 7 represented “strongly agree.” “The scale demonstrated good validity and reliability in the current study. The confirmatory factor analysis revealed an acceptable fit: *χ^2^/df* = 1.448, RMSEA = 0.06, SRMR = 0.023, CFI = 0.998, TLI = 0.995, and Cronbach’s *α* = 0.91.

#### 4.2.4 Constructive conflict coping style

The conflict coping style questionnaire (Han, 2015) was revised using the reverse translation method. A total of 21 items were included in the final questionnaire. It includes two problem-solving strategies: the destructive conflict coping style (exit and neglect) and the constructive conflict coping style (voice and loyalty). ‘Loyalty’ manifests as passive yet constructive, meaning staying put, viewing problems optimistically, and believing that issues will resolve themselves naturally. ‘Voice’ manifests as proactive and constructive, meaning actively discussing conflict issues with partners, reflecting on oneself, and seeking all possible ways to resolve conflicts. ‘Exit’ manifests as proactive and destructive, meaning actively taking actions such as breaking up, leaving the other person, or engaging in physical abuse or violence. ‘Neglect’ involves distancing oneself from the partner, showing a cold attitude, and avoiding communication, which has a significant adverse impact on the relationship. The voice and loyalty subscales were used to measure constructive conflict coping styles, with seven items and four items, respectively, while the exit and neglect subscales were used to measure destructive conflict coping styles, with seven items and three items, respectively. The participants rated the items on a 9-point scale, with 1 to 9 representing the increasing frequency of behavior. The scale demonstrated good validity and reliability in the current study. According to the design of the study, data from the voice and loyalty subscales were selected to assess the reliability and validity of the scale. The confirmatory factor analysis of the voice scale revealed an acceptable fit: *χ^2^/df* = 3.782, RMSEA = 0.075, SRMR = 0.058, CFI = 0.993, TLI = 0.975, and Cronbach’s *α* = 0.82. The confirmatory factor analysis of the loyalty scale revealed an acceptable fit: *χ^2^/df* = 2.95, RMSEA = 0.077, SRMR = 0.047, CFI = 0.99, TLI = 0.940, and Cronbach’s *α* = 0.842.

### 4.3 Statistical analysis

All statistical analyses were conducted using SPSS 25.0 (IBM Corp, Armonk, NY, USA). First, description statistical analysis was performed for the main study variables, and the relationships between the variables were analyzed using Pearson’s correlation analysis. Then, PROCESS macro for SPSS 3.3 (Hayes, 2018), which was developed and is widely used to test complex models with moderating and mediating effects, was adopted to test the hypothesized moderated mediation model with 5,000 bias-corrected bootstrapped samples from the original data. A 95% bias-corrected confidence interval excluding 0 indicated a significant mediation effect. Specifically, Model 58 was used to test the integrated model, with attachment anxiety as the mediator and constructive conflict coping styles as the moderator.

## 5 Results

Table 1 presents the means and standard deviations of the variables, as well as the Pearson’s correlation results among the main research variables. Partner phubbing was negatively correlated with relationship satisfaction and attachment anxiety and positively correlated with loyalty. Relationship satisfaction was negatively correlated with attachment anxiety and positively correlated with voice. Attachment anxiety was positively correlated with voice and loyalty.

**Table 1:** Means, standard deviations, and correlation results.

Variables	M	SD	1	2	3	4	5	6	7
1. Age	21.02	1.93	-						
2. Gender	0.78	0.41	−0.026	1					
3. Partner phubbing	2.75	0.58	0.090^**^	−0.091^**^	1				
4. Relationship satisfaction	3.75	0.84	0.018	−0.124^**^	−0.105^**^	1			
5. Attachment anxiety	3.62	1.20	−0.038	−0.015	0.182^**^	−0.107^**^	1		
6. Voice	6.25	1.55	0.012	0.035	−0.042	0.329^**^	0.079^*^	1	
7. Loyalty	4.30	1.58	0.043	−0.223^**^	0.131^**^	0.019	0.420^**^	0.158^**^	1

*p < 0.05, **p < 0.01, , ***p < 0.001

Then, the hypothesized moderated mediation model was tested using Hayes’ PROCESS macro for SPSS with 5,000 bootstrapped samples (Hayes, 2013). Gender and age were included in the analysis as control variables. The simple mediating model analysis showed that the total effect of partner phubbing on relationship satisfaction was −0.151 [Boot SE = 0.05; Boot 95% CI = (−0.249, −0.054)] and that the indirect effect of attachment anxiety was −0.024 [Boot SE = 0.011; Boot 95% CI = (−0.048, −0.005)], which accounted for 15.89% of the total effect.

The main results of the moderated mediation model analysis consisted of two parts: the regression analysis model and the conditional indirect effect analysis, which are presented in Tables 2–5, respectively.

**Table 2:** The regression analysis of the moderated mediating model (Loyalty).

Dependent variable	Independent variable	*R^2^*	*F*	*β*	*Bootstrap LLCI*	*Bootstrap ULCI*	*t*
Attachment anxiety	Gender	0.205	42.88^***^	0.263 (0.092)	0.082	0.444	2.852^**^
	Age			−0.042 (0.019)	−0.079	−0.004	−2.158^*^
	Partner phubbing			0.288 (0.065)	0.161	0.415	4.455^***^
	Loyalty			0.322 (0.024)	0.275	0.370	13.268^***^
	Partner phubbing× Loyalty			0.0003 (0.031)	−0.060	0.991	0.0114
Relationship satisfaction	Gender	0.047	6.798^***^	−0.253 (0.072)	−0.394	−0.113	−3.544^**^
	Age			0.008 (0.015)	−0.022	0.037	0.523
	Partner phubbing			−0.157 (0.05)	−0.256	−0.058	−3.112^**^
	Attachment anxiety			−0.072 (0.027)	−0.125	−0.019	−2.684^**^
	Loyalty			0.037 (0.021)	−0.004	0.078	1.752
	Attachment anxiety × Loyalty			−0.029 (0.012)	−0.052	−0.007	−2.538^*^

*p < 0.05, **p < 0.01, *** *p* < 0.001.

**Table 3:** The conditional indirect effect analysis (Loyalty).

The level of loyalty	Effect	BootSE	BootLLCI	BootULCI
M − SD	−0.0076	0.0121	−0.0346	0.0149
M	−0.0208	0.0103	−0.0442	−0.0041
M + SD	−0.0341	0.0134	−0.0642	−0.0111

**Table 4:** The regression analysis of the moderated mediating model (Voice).

Dependent variable	Independent variable	*R^2^*	*F*	*β*	*Bootstrap LLCI*	*Bootstrap ULCI*	*t*
Attachment anxiety	Gender	0.045	7.758^***^	−0.002 (0.099)	−0.196	0.192	−0.018
	Age			−0.035 (0.021)	−0.076	0.007	−1.655
	Partner phubbing			0.399 (0.071)	0.260	0.538	5.632^***^
	Voice			0.070 (0.026)	0.018	0.121	2.649^**^
	Partner phubbing × Voice			0.032 (0.038)	−0.043	0.107	0.836
Relationship satisfaction	Gender	0.159	6.798^***^	−0.302 (0.065)	−0.431	−0.174	−4.626^***^
	Age			0.006 (0.014)	−0.022	0.033	0.405
	Partner phubbing			−0.123 (0.047)	−0.215	−0.030	−2.590^**^
	Attachment anxiety			−0.084 (0.023)	−0.1294	−0.0393	−3.676^***^
	Voice			0.172 (0.018)	0.1372	0.2076	9.6193^***^
	Attachment anxiety × Voice			−0.033 (0.013)	−0.058	−0.009	−2.653^**^

*p < 0.05, **p < 0.01, *** *p* < 0.001.

**Table 5:** The conditional indirect effect analysis (Voice).

The level of voice	Effect	BootSE	BootLLCI	BootULCI
M - SD	−0.0114	0.0140	−0.0427	0.0137
M	−0.0337	0.0123	−0.0607	−0.0129
M + SD	−0.0611	0.0228	−0.1111	−0.0214

First, as shown in Tables 2, 4, partner phubbing was positively associated with attachment anxiety, while attachment anxiety was negatively associated with relationship satisfaction. Partner phubbing was also significantly associated with relationship satisfaction. These results indicated that attachment anxiety could partially mediate the association between partner phubbing and relationship satisfaction. Meanwhile, the interaction effect of attachment anxiety and loyalty was significant, highlighting the moderating role of loyalty in the association between partner phubbing and relationship satisfaction. In addition, the interaction effect of attachment anxiety and voice was also significant, highlighting the moderating role of voice in the association between partner phubbing and relationship satisfaction.

**Figure 2:**
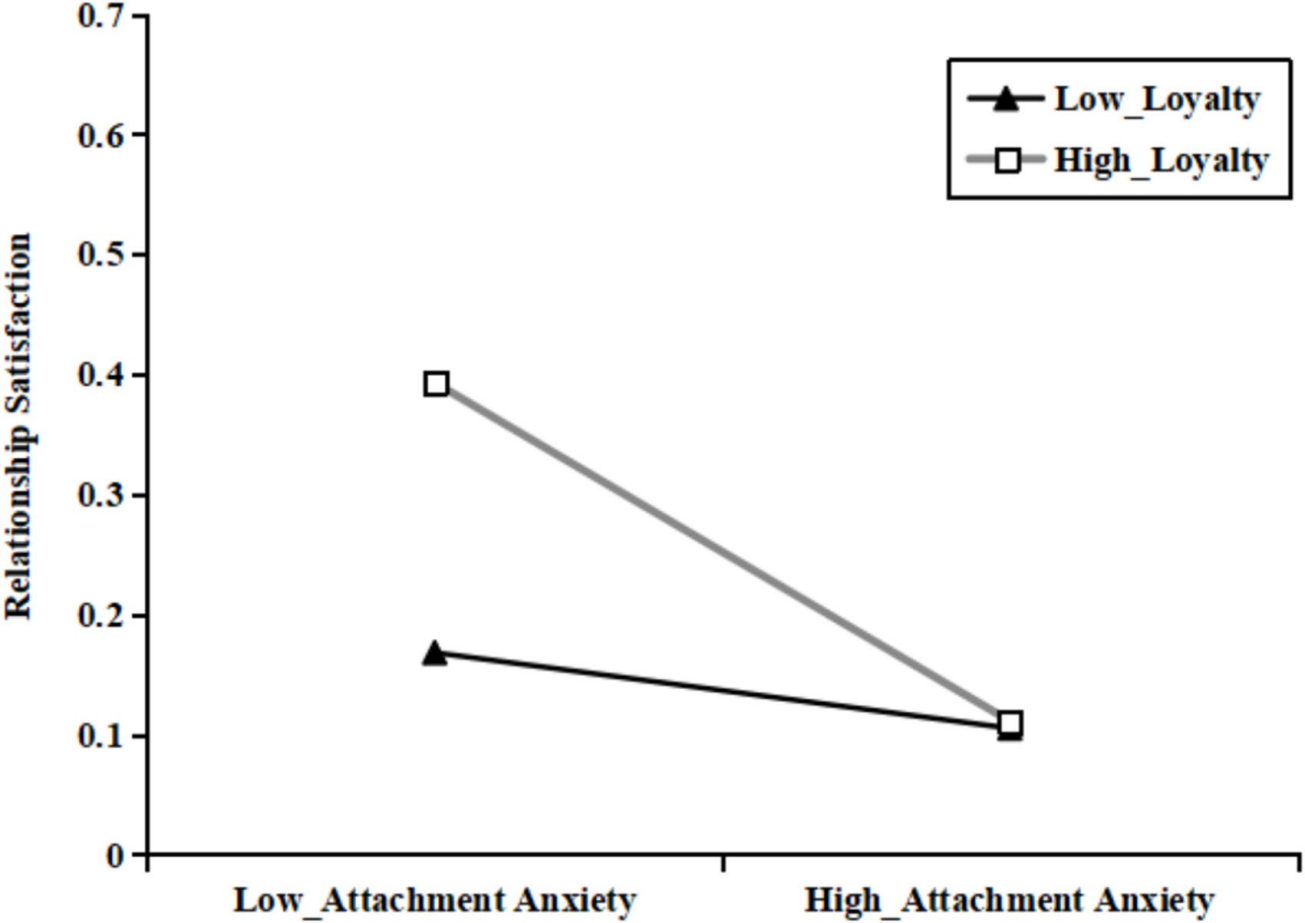
The association between attachment anxiety and relationship satisfaction at different levels of loyalty.

**Figure 3:**
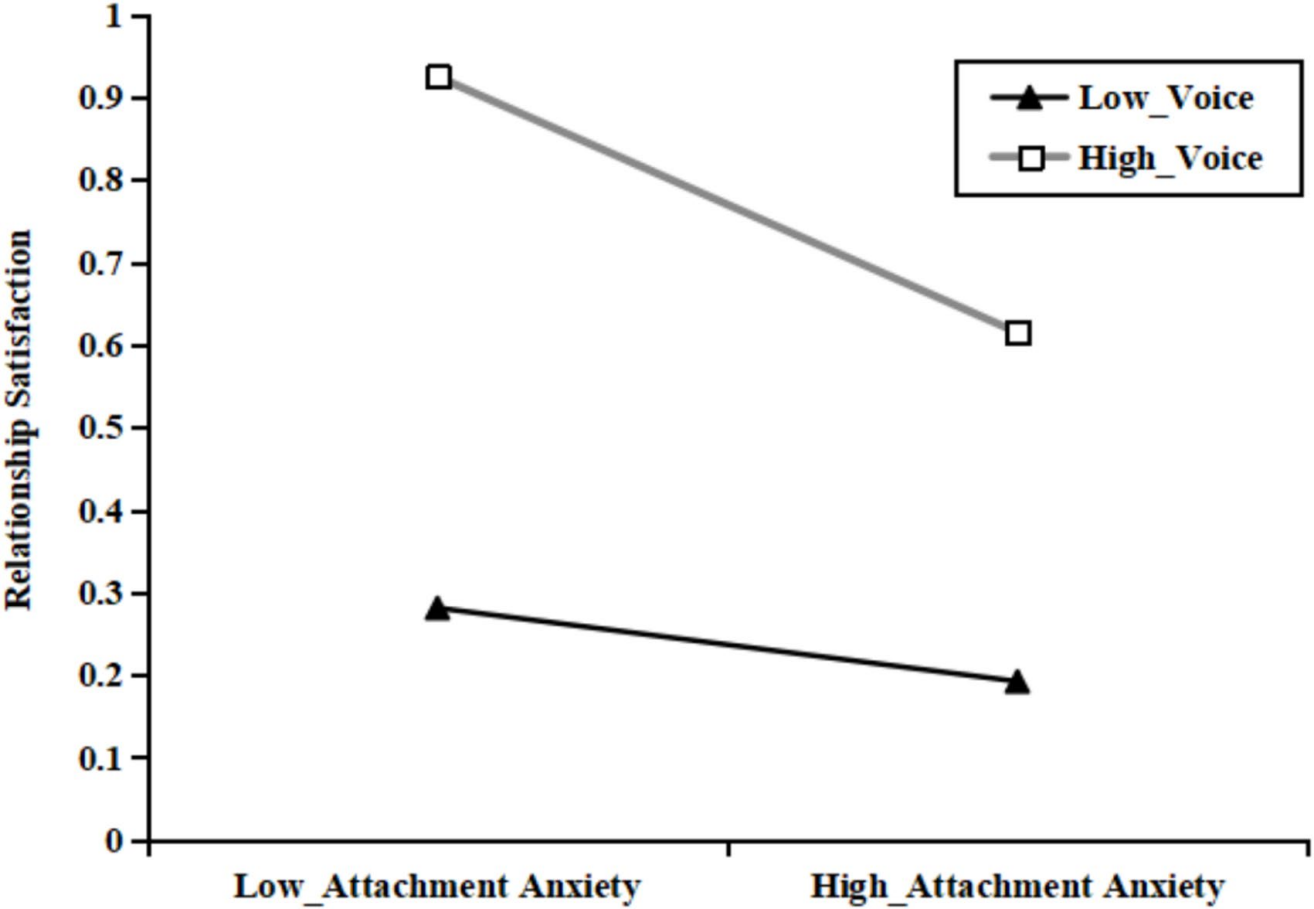
The association between attachment anxiety and relationship satisfaction at different levels of voice.

Then, as shown in Tables 3, 5, all three mediating effects (at the mean of loyalty or voice, as well as one standard deviation above and below the mean of loyalty or voice) were examined. Among these, the mediating effects at loyalty_M - SD_ and voice_M - SD_ were not significant and included zero. Specifically, the indirect effect of partner phubbing on relationship satisfaction through attachment anxiety was observed among the individuals with different levels of loyalty or voice. However, the mediating effect of attachment anxiety was stronger for the individuals with higher levels of loyalty or voice (see Figures 2, 3).

## 6 Discussion

This study examined the psychological mechanisms underlying the relationship between young adult partner phubbing and relationship satisfaction. First, the results revealed that young adult partner phubbing was negatively associated with relationship satisfaction, which is consistent with previous findings on the “interference of technology in couple relationships” (Mcdaniel and Coyne, 2016). Phubbing among young people is primarily driven by fear of missing out, intolerance of uncertainty, and problematic social media use (Cheng et al., 2022). The presence of cell phones can interfere with relationship satisfaction among young romantic partners, as the basic human needs for control and attachment are compromised when individuals perceive their partners as mentally absent (Roberts and David, 2016). According to interdependence theory, one partner’s emotions, cognition, or behavior can influence the other partner’s emotions, cognition, or behavior, creating a reciprocal effect that continues to shape the relationship (Rodriguez et al., 2014). This theory suggests that the level of interdependence between partners—the extent to which each partner relies on or needs the relationship—characterizes the relationship dynamics (McNulty and Karney, 2002). For example, in contemporary Chinese couples, wives often balance family caregiving responsibilities with contributions to the household economy. When husbands indulge in smartphone use and neglect their spouses’ psychological needs, wives may feel less satisfied in their marriage (Zhang et al., 2023). In contrast, young romantic partners tend to prioritize psychological interdependence over reciprocity, making them particularly vulnerable to the detrimental effects of phubbing on relationship satisfaction.

A second goal of this study was to identify potential mediators that explain the underlying mechanisms between young adult partner phubbing and relationship satisfaction. A deeper understanding of these mechanisms could inform interventions to mitigate the negative effects of phubbing. While several studies have explored mediating variables between phubbing and relationship satisfaction, fewer have focused specifically on young adult romantic partners (Beukeboom and Pollmann, 2021). Evidence suggests that partner attachment significantly predicts relationship satisfaction (Ben-Ari and Lavee, 2005). Using mediation models, this study found that attachment anxiety mediates the link between young adult partner phubbing and relationship satisfaction. The zero-order correlations (Table 1) showed that young adult partner phubbing was positively related to attachment anxiety, and both were negatively related to relationship satisfaction. Furthermore, attachment anxiety consistently mediated the relationship between phubbing and relationship satisfaction across different responses (Tables 2, 3). Previous studies have shown that the perceived reward of the initiator plays a significant role in shaping the target’s reactions when expectations are met or unmet (Burgoon, 2015). Partner phubbing reduces an individual’s sense of security and emotional support, exacerbating psychological distress, such as anxiety (Slatcher and Selcuk, 2017). Phubbing increases feelings of worry and uncertainty in individuals while neglecting their partner’s emotional needs. In addition, romantic partners who frequently engage in phubbing are more likely to experience conflicts related to their spouse’s phone use (Togar et al., 2023). In particular, when one partner engages in more obvious phubbing, it can cause anxiety in another partner, which, in turn, reduces the stability of the partner’s relationship. This finding also aligns well with the key points of independence theory (Kelley et al., 2023), which argues that the behavior of one partner is influenced by both themselves and their partner. In this study, it was observed that attachment anxiety serves as an effective individual protection mechanism for young adults experiencing partner phubbing, as it generates an emotional response to the neglected partner’s behavior and further impacts romantic relationships. Thus, young adult partner phubbing was negatively associated with relationship satisfaction through the mediating role of attachment anxiety. It is worth noting that the relationship between young adult partner phubbing and relationship satisfaction is particularly strong, as young adult partners are at higher risk of phubbing and experience its effects more intensely. This relationship should be further explored in future studies. Moreover, this finding extends interdependence theory, suggesting its applicability to romantic relationships.

Furthermore, the current results highlighted the significant moderating role of constructive conflict coping styles in the relationship between attachment anxiety and relationship satisfaction. The simple slope analysis indicated that the negative indirect effect of attachment anxiety on relationship satisfaction was stronger for the individuals with high levels of constructive conflict coping styles (e.g., voice or loyalty) compared to those with low levels. When the partners exhibited high levels of voice or loyalty, attachment anxiety had a strong negative effect on relationship satisfaction; however, this effect was attenuated to non-significance when the levels of voice or loyalty were low. These results are relatively consistent with the findings of similar studies (Roberts and David, 2023). Compared to loyalty, voice was more effective in moderating the mediation role of attachment anxiety. When attachment anxiety leads to feelings of unacceptance and neglect, it can exacerbate psychological distress. In other words, perceived loyalty alone may not effectively maintain relationship satisfaction at higher levels of attachment anxiety. When young adult partners adopt a voice coping style, they can effectively regulate negativity and enhance feelings of security, thereby improving relationship satisfaction (Stanton et al., 2019). Therefore, fostering positive perceptions between partners can help mitigate the erosion of relationship satisfaction caused by attachment anxiety. This finding further elucidates the mechanism underlying young adult partner relationship satisfaction by emphasizing the role of individual differences.

## 7 Implications and limitations

We are in the digital era, and our individual behavior is influenced by digital technology. This study found that attachment anxiety mediates the relationship between young adult partner phubbing and relationship satisfaction and that constructive conflict coping styles moderate the relationship between attachment anxiety and relationship satisfaction. These findings provide valuable insights into the psychological mechanisms underlying partner phubbing and offer practical implications for improving relationship satisfaction among young adults. First, this study deepens our understanding of young adult partner relationship satisfaction and phubbing. Second, these findings clarify the unique psychological mechanism of phubbing in young romantic partners. Thirdly, these results also provide practical implications for resolving the conflicts caused by phubbing. For example, attachment anxiety could reduce the relationship satisfaction of young romantic partners. However, when young romantic partners adopt constructive conflict coping methods (such as loyalty or voice), these methods could effectively alleviate the negative effects of attachment anxiety on relationship satisfaction. Finally, this study also extends research on interdependence theory and expectancy violations theory, suggesting that they could explain the behaviors of young romantic partners in virtual and real-life interactions, especially in the case of young romantic partner phubbing.

This study has several limitations. First, due to the cross-sectional method and the relatively small sample size, a causal inference could not be effectively drawn. Future research should adopt a longitudinal research design to confirm the causality of this relationship in a larger sample. Second, the rate of romantic relationships or marriages among young Chinese people is relatively low, and the establishment of intimate relationships is also delayed. This is mainly due to the low willingness of contemporary young Chinese people to engage in romantic relationships, suggesting that future studies should consider the influence of willingness to form relationships or the strength of intimacy. Third, this study only considered the effect of partner phubbing on relationship satisfaction from the perspective of a single young romantic partner. Future studies should consider using the Actor–Partner Interdependence Model and explore the potential mechanisms of the mutual influence of phubbing among young couples on relationship satisfaction. Finally, it is necessary to classify different types of young adult partner phubbing, as different types of partners may have varying effects on behavioral outcomes, which would further deepen our understanding of this issue. In addition, phubbing is primarily measured based on peer assessment, and future studies could consider other measures (e.g., individual mobile phone use time or the interaction time of the partner) to address the shortcomings of the subject-assessment results.

## Data Availability

The original contributions presented in the study are included in the article/Supplementary material, further inquiries can be directed to the corresponding author.
