# Supplementary material for: Young adult partner phubbing and relationship satisfaction: the mediating role of attachment anxiety and the moderating role of constructive conflict coping style
Source: Front Psychol. 2025 Feb 25;16:1490363. doi: 10.3389/fpsyg.2025.1490363 (PMC11893583; doi:10.3389/fpsyg.2025.1490363)
Supplement: Supplementary file 1 [file Data_Sheet_1.PDF]

Paper discussion & revision:
Young Partner Phubbing and Relationship Satisfaction: The Mediating Role of Attachment Anxiety and the Moderating Role of Constructive Conflict Coping Style

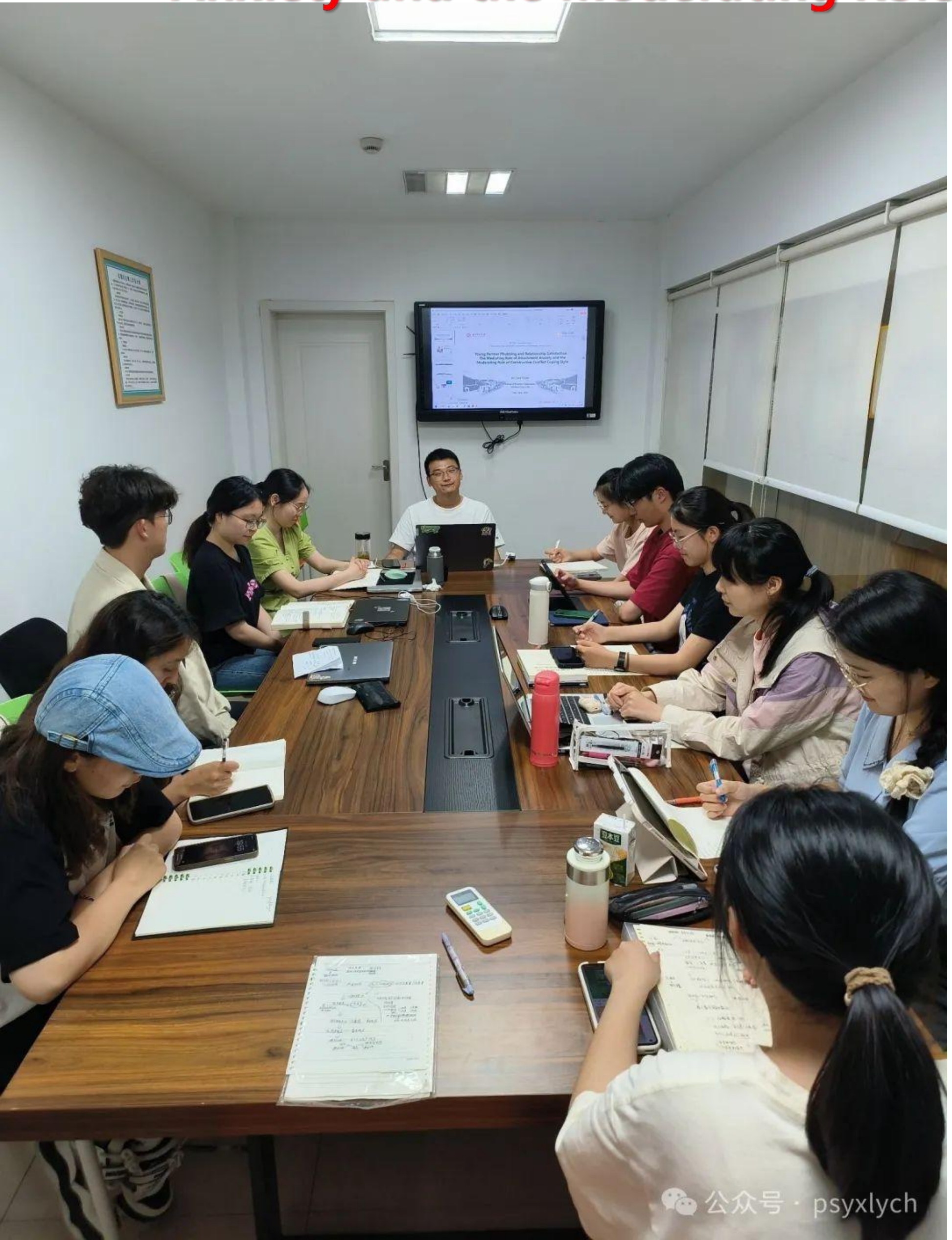

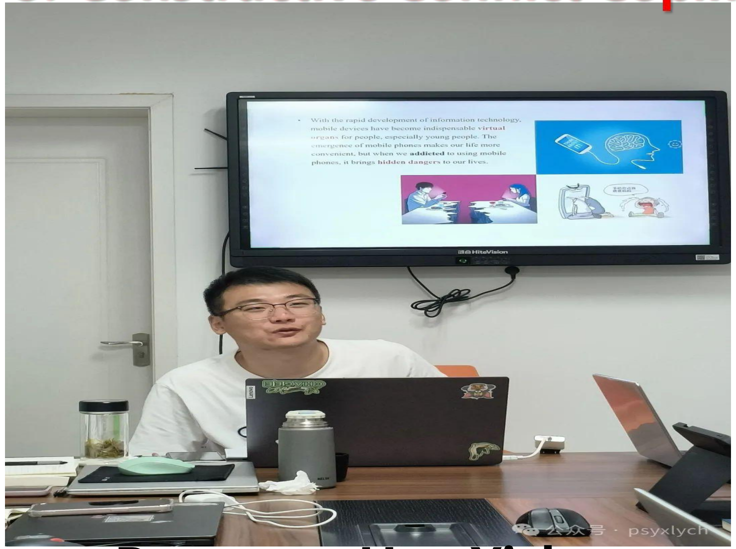

Reporter: Han Yichu

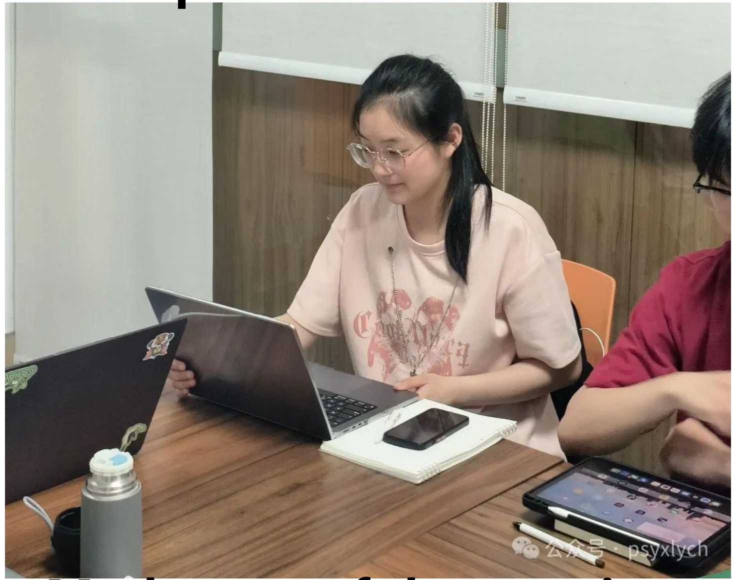

Moderator of the meeting: He Yifan

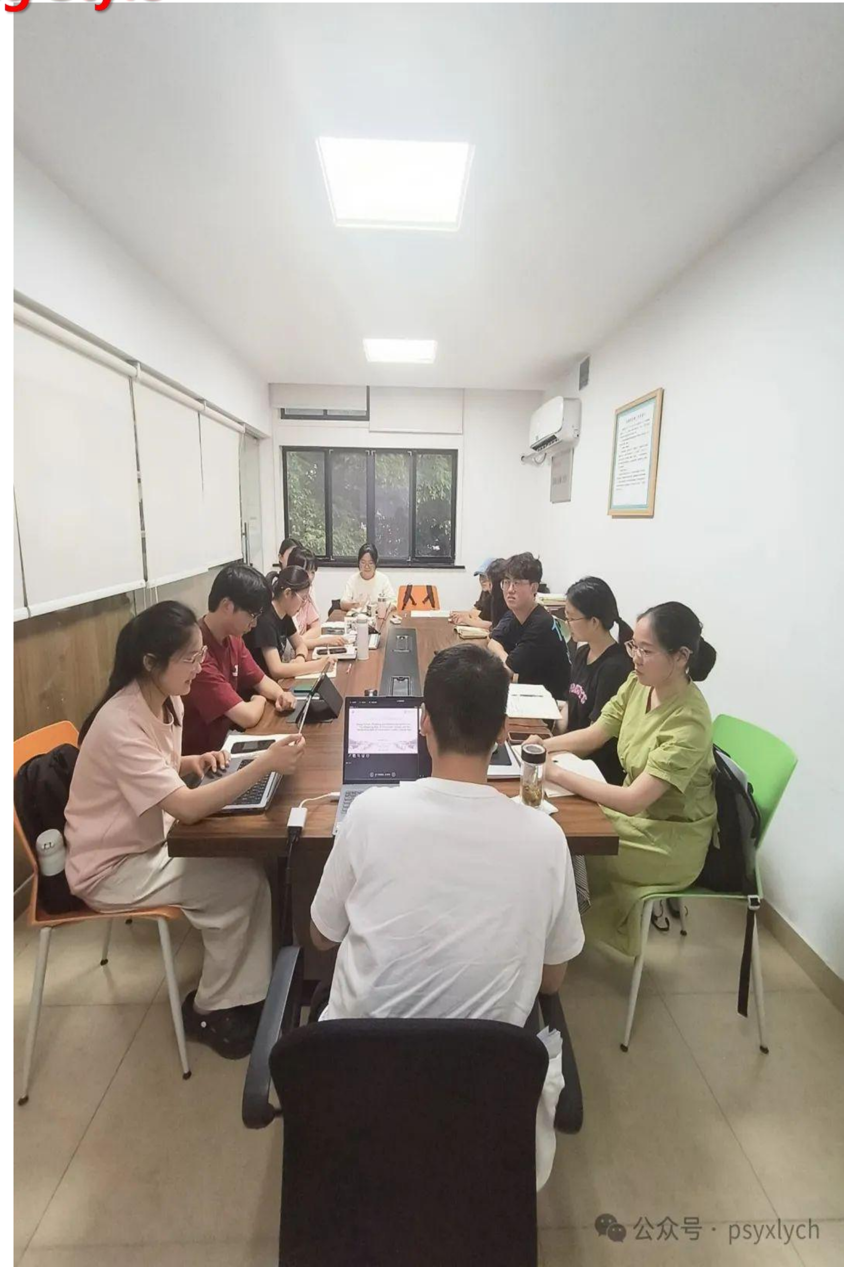

湖州师范学院
Huzhou University

Han Lab
Dream of Hi π，Flow with Happy

Yi Chu's Academic Views
The second phase of the Hi π supervisor's achievement report activity

Young Partner Phubbing and Relationship Satisfaction: The Mediating Role of Attachment Anxiety and the Moderating Role of Constructive Conflict Coping Style

Dr. Han Yichu

**School of Teachers Education,
Huzhou University**

May 20th, 2024

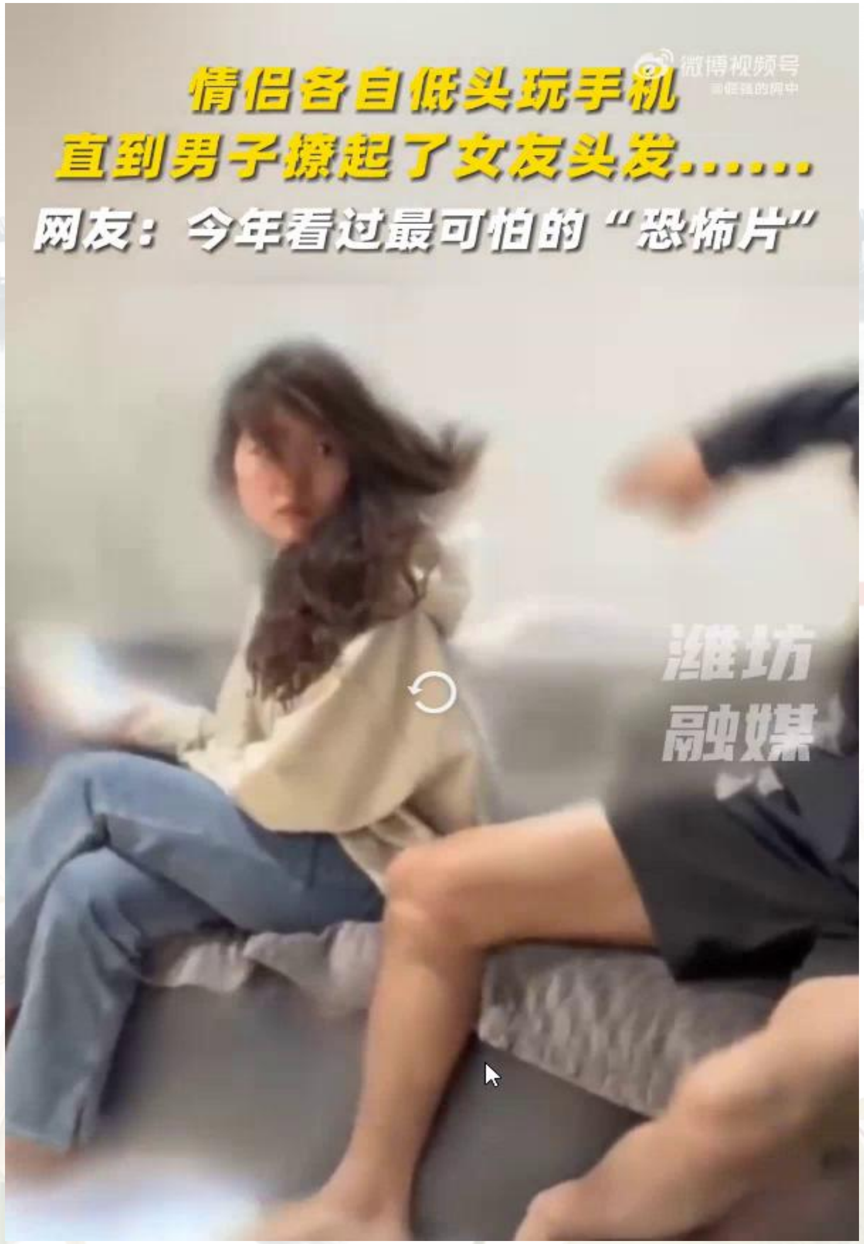

Young Partner Phubbing

&

Relationship Satisfaction

Content

No.1 Introduction

No. 2 Literature Review

No. 3 Materials and Methods

No. 4 Results

No. 5 Discussion

Han Lab

Dream of Hi π, Flow with Happy

No.1 Introduction

- With the rapid development of information technology, mobile devices have become indispensable **virtual organs** for people, especially young people. The emergence of mobile phones makes our life more convenient, but when we **addicted** to using mobile phones, it brings **hidden dangers** to our lives.

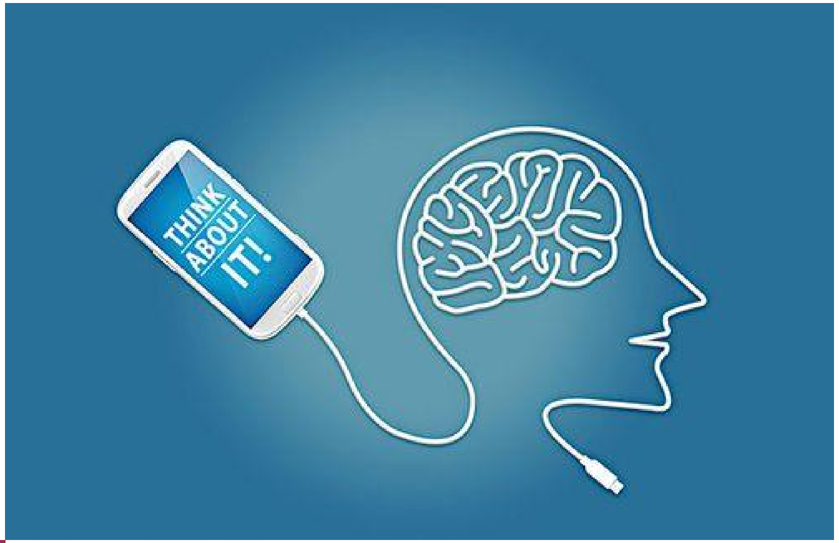

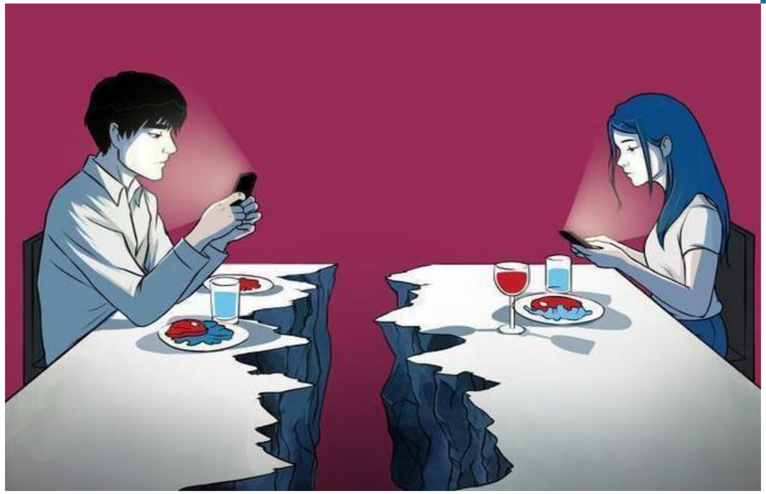

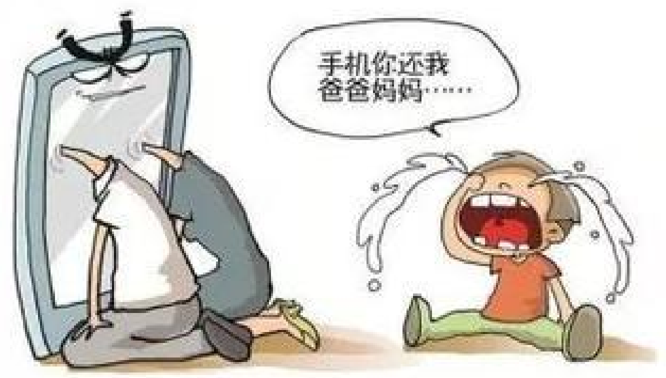

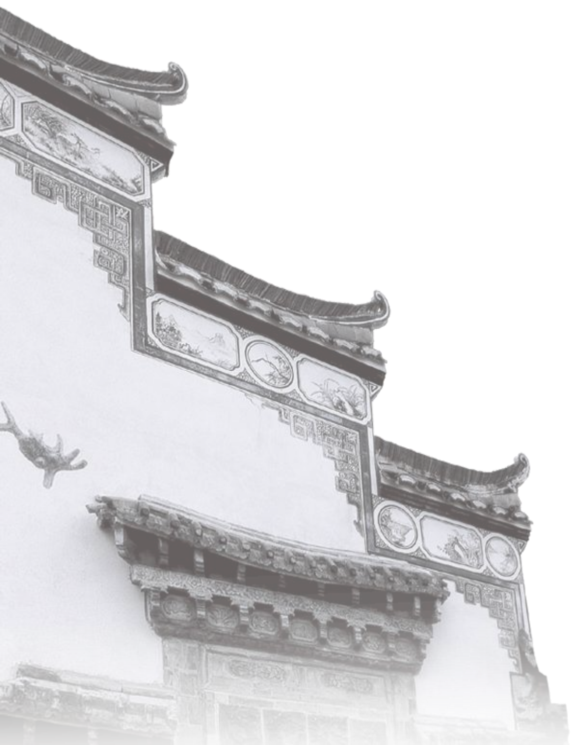

- **Phubbing** is a brand new expression and stands for "phone snubbing", describes the act of snubbing someone in a social setting by looking at your phone instead of paying attention (Ugur & Koc, 2015). When this phenomenon occurs in young couples, it is called **young partner phubbing**.
- In fact, young partner phubbing is that both couples are **present together**, but one party is **mentally absent** due to the abuse of mobile phone, which is an **interactive behavior**, not a one-way behavior. Partner relationships perceived as **“indifferent”** may be particularly distressing, with subsequent downstream consequences for health(Ross, et al., 2018).

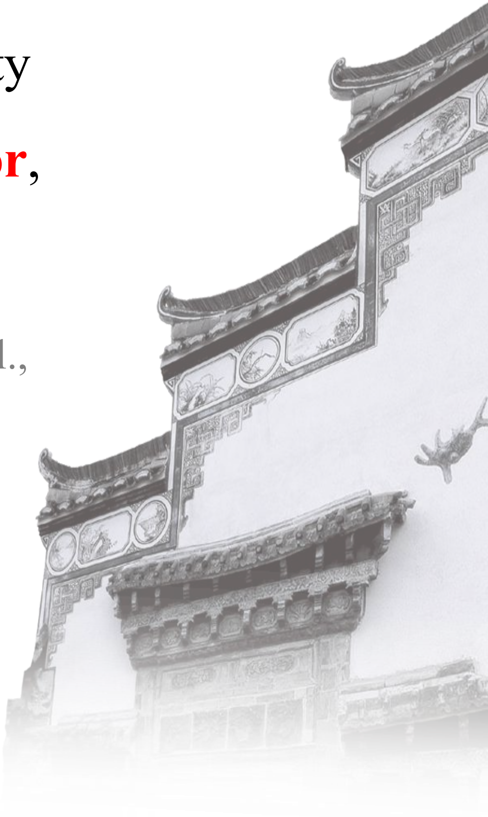

indifferent， 漠不关心

- Using Smartphones and tablet PC has seriously affected interpersonal interactions among individuals, especially for the **youth partners**. Smartphones can **decrease** the **quality** of interpersonal interactions(Dwyer et al., 2018). Mobile internet devices directly lead to the isolation between people and make people lack the necessary communication. Although Smartphones can meet the speed, quality and effectiveness required for communication, their dependence on smartphones can lead to psychological barriers, such as phubbing. It **distances** relationship between friends and family member(Anshari et al., 2016). The user group of smart phones is **mainly** **young people**, so phubbing is common among them. In fact, technology devices (such as computers, cell or smartphones, or TV) frequently **interrupted** partners' interactions. Partner's relationship is full of **conflict**,more **depressive** symptoms, and **lower life satisfaction**(Mcdaniel & Coyne, 2016).

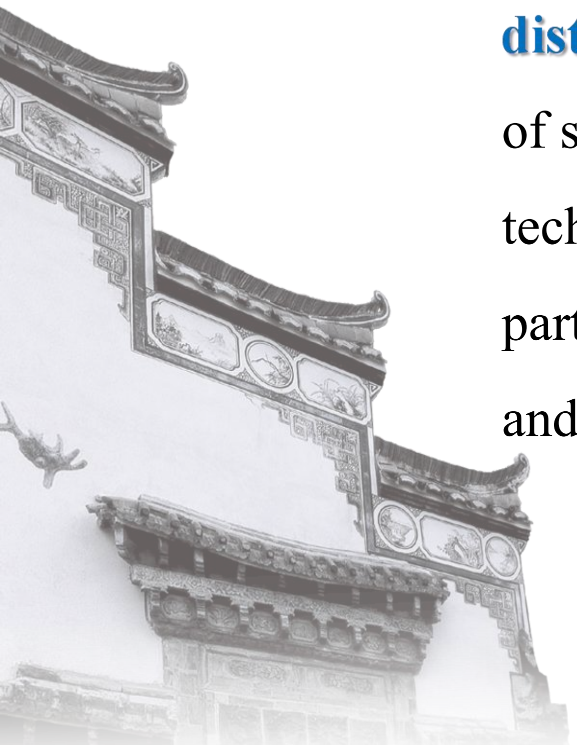

e.g. absent present during partner communication may arouse suspicion and fear in spouses, which in turn escalates neglect into conflict.

Exacerbate marital conflicts and anxiety

Young partner phubbing may exacerbate marital conflicts and anxiety

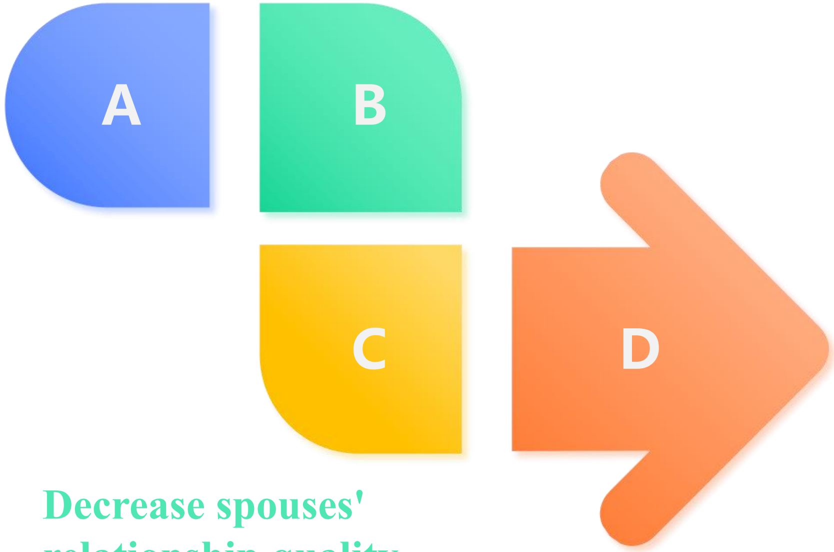

Young partner phubbing has been a new negative trigger

Decrease spouses' relationship quality and satisfaction

e.g. due to the lack of necessary communication, the communication mechanism between the couples is blocked, which may seriously lead to the breakdown of the love relationship.

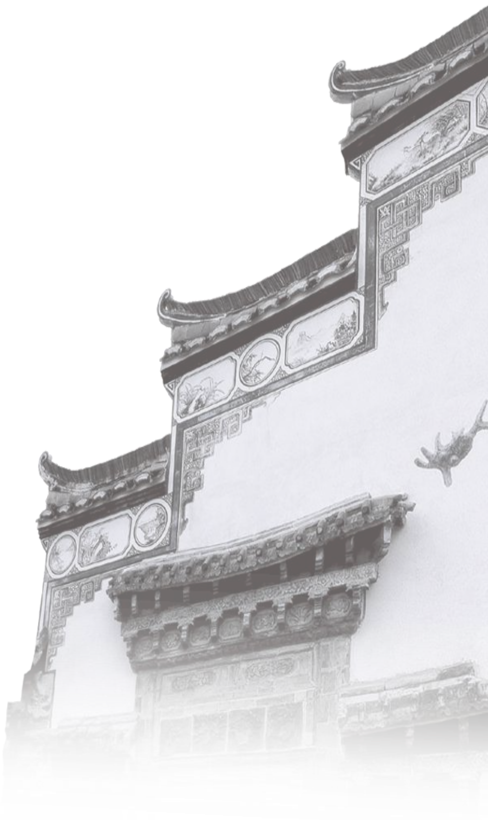

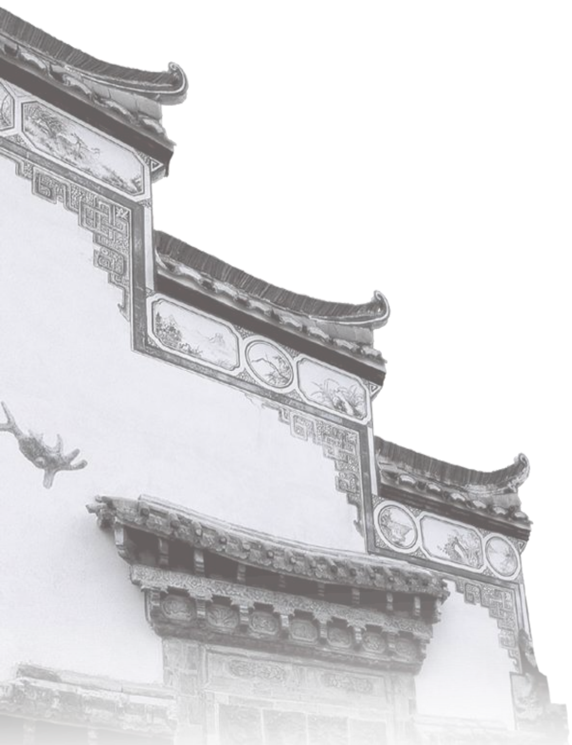

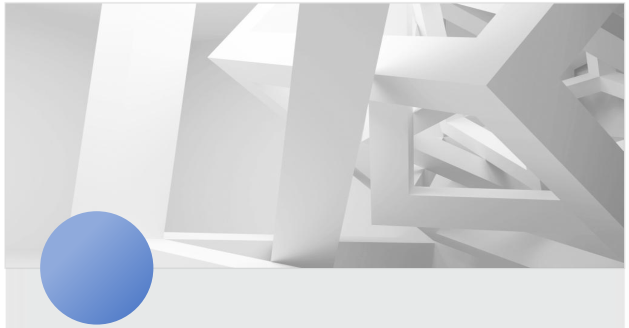

Previous studies

have paid more attention to the occurrence and harm of phubbing at the individual level(Karadağ et al., 2015).

Some studies have demonstrated that shared phones can mitigate the adverse effects of phubbing from a positive conflict resolution approach(Beukeboom & Pollmann, 2021), and the mobile phone exclusion effect caused by phubbing may threaten the basic psychological needs (especially the sense of belonging)(Chotpitayasunondh & Douglas, 2018).

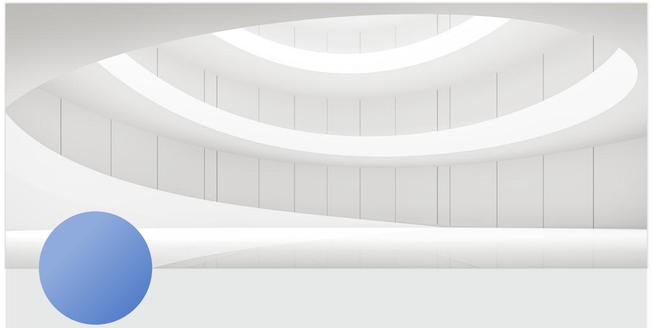

Present research

was to examine the effect of young partner phubbing on relationship satisfaction and the potential mediating role of attachment anxiety and the moderating role of constructive conflict coping style.

No.2

Literature Review

Han Lab

2.1 Partner Phubbing and Relationship Satisfaction

Negative

Partner phubbing is negatively related to relationship satisfaction. (Beukeboom & Pollmann, 2021; David & Roberts, 2021)

zero

Partner phubbing was not significantly associated with relationship satisfaction. (Cizmeci, 2017; Wang et al., 2021)

- Although the previous studies have not yet reached an agreement, the phubbing phenomenon hinders personal relations, **possibly altering the fabric of social interactions**(Agata, 2021).
- **Intimacy** thus develops in **interactions** in which one individual discloses personal information, thoughts, and feelings to a partner, receives a response from the partner, and interprets that response as understanding, validating, and caring(Laurenceau et al., 1998). Partner phubbing may **hinder** the positive interaction between one partner or both, leading to **cracks** in the intimate relationship.

2.1 Partner Phubbing and Relationship Satisfaction

- Phubbing is essentially a **subtype** of **social exclusion**, formed based on the abuse of **digital technology devices** (e.g. tablets, mobile phones, VR, et al.).

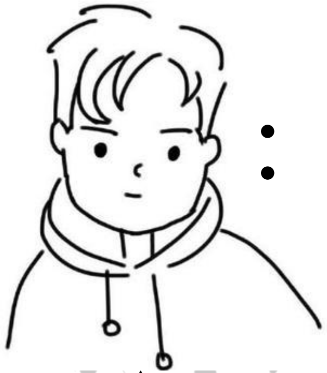

- keep their heads down to play with their mobile phones
- the lack of eye contact with their partners

(Nakamura,2015)

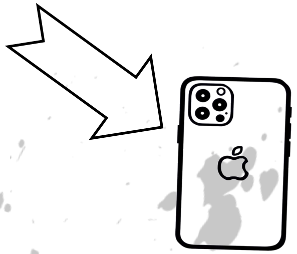

Young partners are more **flexible with emerging technologies** than older, and their relationships are **relatively fragile**.

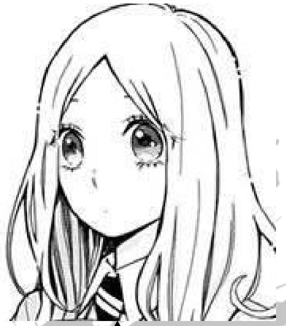

- feels lower intimacy
- reducing partner relationship satisfaction

(Halpern & Katz, 2017)

trigger negative feedback

(Negative feedback from partner phubbing is more likely to **hinder** the expectation of youth partners from reaching positive relationships with each other.)

2.2 Attachment Anxiety as a Mediator

- The romantic partner's attachment mainly refers to the mutual assistance and attachment between romantic partner, as well as the sense of security and belonging brought by it(Lu et al., 2009).

  - relationship needs
  - dependence on intimacy
  - lower sense of self-worth
  - high levels of negative emotions

attachment anxiety

attachment avoidance

adults demonstrate reliable individual differences. (Crowell et al., 2016)

2.1 Partner Phubbing and Relationship Satisfaction

The relationship of partner phubbing and relationship satisfaction is also affected by **self-esteem** and the **marital status**(Wang et.al., 2021).

- **Low self-esteem individuals** usually assume that their partner sees them **in the same negative way** as they do(Murray et.al., 2000). Whether partner phubbing affects relationship satisfaction is influenced by **individual subjective feelings**, including both **emotional responses** triggered by **negative feedback** and individual subjective evaluation.

Cizmeci's (2017) study that indicates there was a marginally significant difference in partner vomiting between married and unmarried adults.

Different cognitive mechanisms

- **Young partners** ⇨ more strained about the **emotional problems** of phubbing. (Miller-Ott, A., & Kelly, 2015)
- **Older partners** ⇨ concerned about the **property** of phubbing behavior (such as disloyalty and unfair). (Clayton, 2014)

2.2 Attachment Anxiety as a Mediator

- The romantic partner's attachment mainly refers to the mutual assistance and attachment between romantic partner, as well as the sense of **security** and **belonging** brought by it(Lu, et al., 2019).
- The romantic partner's attachment is a kind of adult attachment including **attachment anxiety** and **avoidance**, and adults demonstrate reliable individual differences (Crowell, et al., 2016)

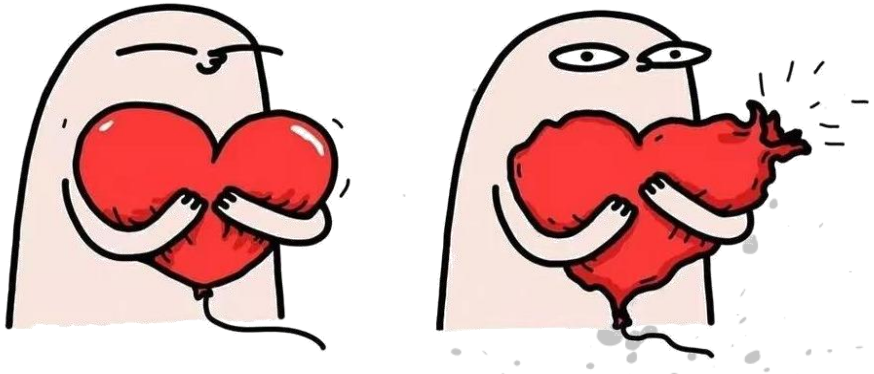

Why choose Attachment Anxiety?

attachment anxiety is more inclined to
- relationship needs
- dependence on intimacy

accompanied by
- a lower sense of self-worth
- high levels of negative emotions

(Shaver & Mikulincer, 2002)

&

attachment anxiety have lower partner relationship satisfaction.
(George & Rholes, 2020)

2.2 Attachment Anxiety as a Mediator

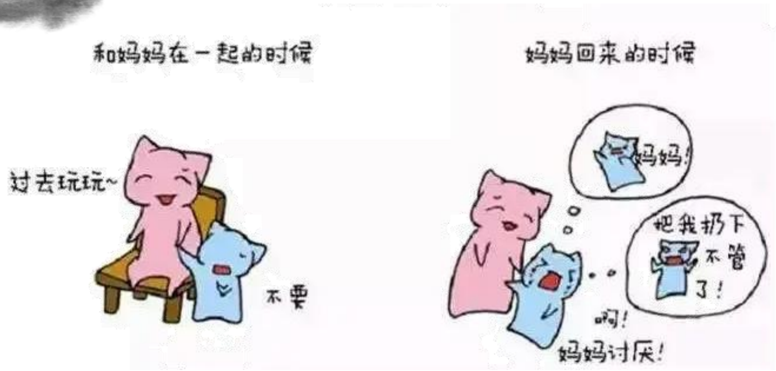

都是童年惹得祸

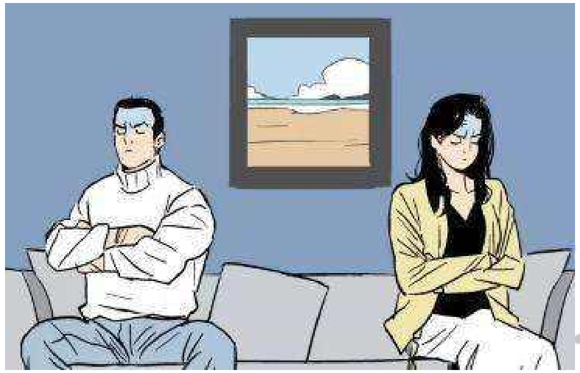

Attachment anxiety did not entirely result from maladaptive romantic relationships, but rather, rooted in negative experiences during childhood.

依恋焦虑并不完全是由于适应不良的浪漫关系，而是根植于童年时期的负面经历。

- The intimate partner behavior have been linked to attachment style, most notably attachment anxiety(Sullivan, et al., 2023).
- A meta-analytic study suggests that attachment anxiety is negatively associated with relationship satisfaction(Candel & Turliuc, 2019).
- Adults reporting higher attachment anxiety scores sought greater positive feedback about their romantic relationships, yet were more willing to incorporate negative feedback into their self-views and more likely to be negatively emotionally affected by such feedback(Carnelley, 2007; Dykas & Cassidy, 2011).

2.2 Attachment Anxiety as a Mediator

- In addition, in a meta analysis of the impact of attachment anxiety and mobile phone addiction, Zhang et al(2022) found that the relationship between attachment anxiety and mobile phone addiction were highly positively associated but not moderated by gender. It seems that the use of media devices such as mobile phones may **activate** and **maintain** the individual attachment anxiety state and response.
- **Attachment theory** postulates that as a result of developmental histories of support seeking that vary in degree of the attainment of support, individuals develop working models or social expectations that influence how they perceive and react to close relationships(Shaver & Mikulincer, 2002).

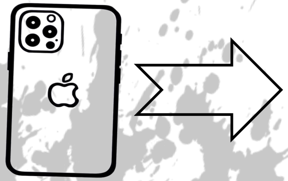

Compensatory Attachment

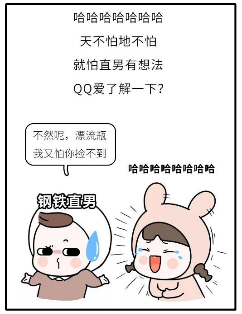

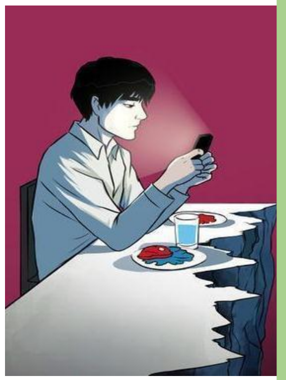

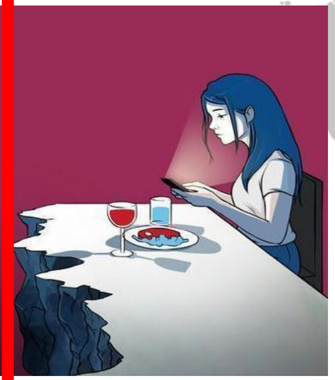

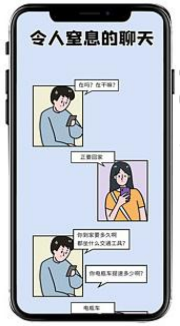

Mentally Absent

2.3 Constructive Conflict Coping Style as a Moderator

- Previous literature has relatively fully demonstrated the relationship between phubbing and romantic relationship satisfaction. However, in the process of interaction between young partners, it is **unclear** how the constructive conflict coping style adopted by young partners works in the conflict caused by phubbing.

- **Coping strategies** can be **described as** the internal resources that individuals employ, which involve their emotional, cognitive, and behavioral efforts to or mitigate stresors in particular situations(Lazarus & Folkman, 1984).

- Previous studies have shown a **significant correlation** between conflict coping style and attachment anxiety, but **not significantly** to predict marital quality(Eva, et al., 2017).

2.3 Constructive Conflict Coping Style as a Moderator

- In the process of contact with a couple, if the other person provides a "vague" support, adults reporting higher attachment anxiety scores will focus on the negative information, which often leads to misunderstanding(Collins & Feeney, 2004).

- There were evidences that the more spouses **respected** and **loved** each other, the **happier** was their marriage(Danesh & Hydarian, 2006).

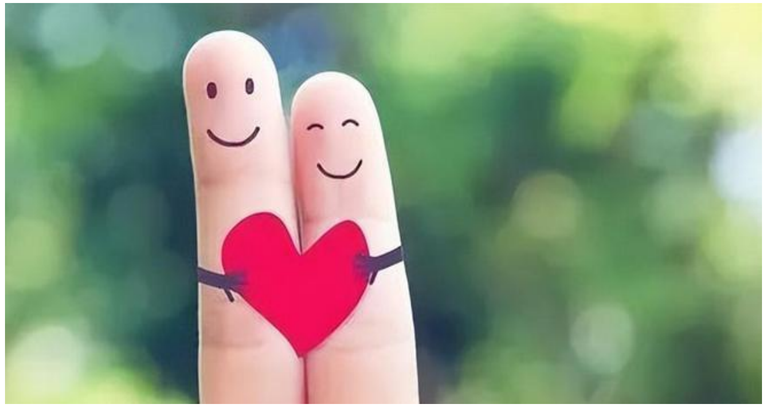

respecte + love = happiness

2.3 Constructive Conflict Coping Style as a Moderator

- As discussed above, romantic relationships were influenced by uncertainty.
- According to the **uncertainty reduction theory**(Berger & Calabrese, 1974), feelings of uncertainty within romantic relationships may prompt people to use passive, active, and interactive strategies to gain information about their partner.

Relevant studies also tested the moderating role of constructive conflict coping style found that

- **loyal** could **moderate** the relationship between attachment anxiety and relationship satisfaction(McDaniel, et al.,2017),
- and **length of the romantic relationship** could**n't moderate** the relationship between social networking site use and disloyalty behavior (Clayton, 2014).

2.4 Research Hypothesis

Based on the previous literature, it was hypothesized that:

- **H1**: Young partner phubbing would be negatively related to relationship satisfaction;
- **H2**: Partner attachment could have a mediating role between Young partner phubbing and relationship satisfaction;
- **H3**: Constructive conflict coping style could moderate the mediating effects of partner attachment, and the mediating effects would be stronger in individuals with high levels of conflict coping style.

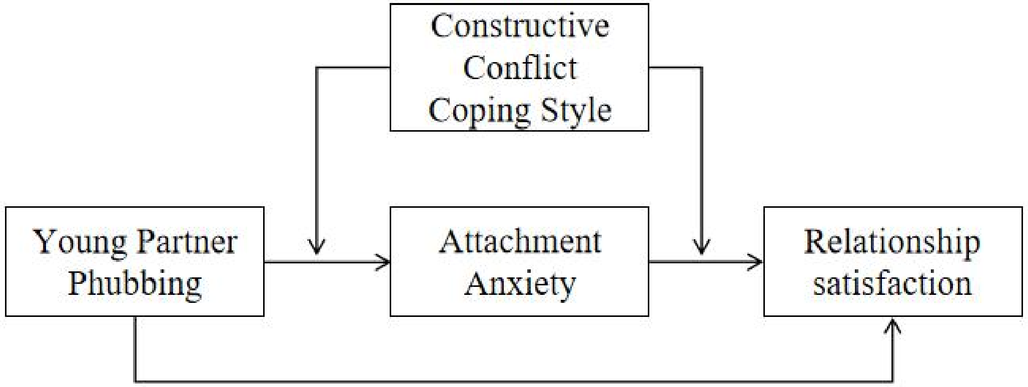

Figure 1. The conceptual model

No.3 Materials and Methods

Participants

- Convenience sampling (***N*=2052**)
- We recruited voluntary participants with a minimum age of 18 years and a **relationship** of at least 6 months.
- After excluding participants who did not fulfill criteria or did not complete the questionnaire, **the final sample consisted of N = 837.**
- female (n = 461, 55.1%)
- male (n = 376, 44.9%)
- **M =21.02 (SD = 1.931)**.

Measurement

Partner Phubbing Scale

(Roberts & David, 2016)

- 9 items
- 5-point scale
- Cronbach's α was **0.746**

Relationship Assessment Scale

(Hendrick, 1988)

- 7 items
- 5-point scale
- Cronbach's α was **0.859**

Chinese Adaptation of ECR Scale

(Li & Kato, 2006)

- 18 items **(Attachment anxiety Scale)**
- 7-point scale
- Cronbach's α was **0.91**

Conflict coping style questionnaire

(Han, 2015)

- 7 items**(voice subscale)**
- 4 items**(loyalty subscale)**
- 9-point scale
- Cronbach's α was **0.842**

Statistical Analysis

SPSS 25.0 (IBM Corp, Armonk, NY, USA).

- Description statistical analysis
- Pearson's correlation analysis.

PROCESS Procedure for SPSS Version 3.3**(Hayes, 2018)**

- 5000 bias-corrected bootstrapped samples from the original data, and 95% bias-corrected confidence intervals excluding 0 had a significant mediation effect.
- **Model 58** was conducted to test the integrated model with attachment anxiety as the mediator, and constructive conflict coping style as the moderator.

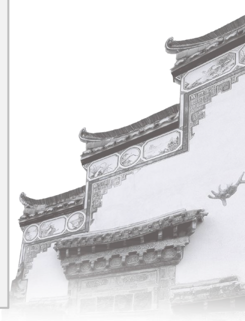

No.4 Results

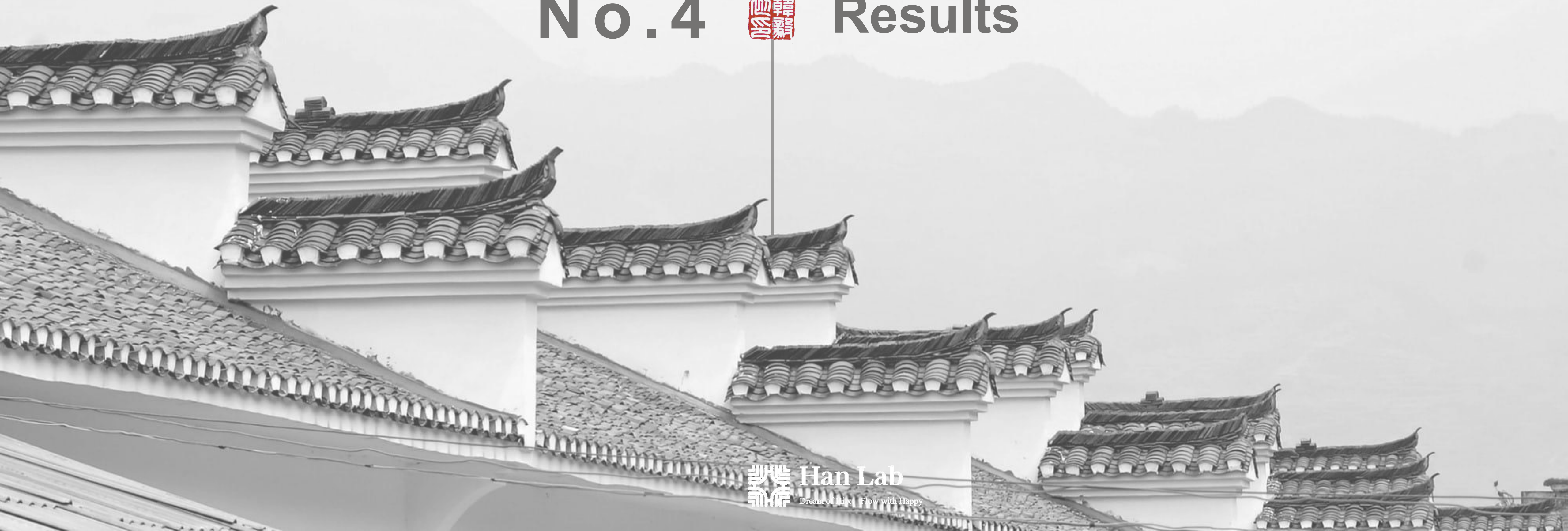

Table 1. Means, standard deviations, and correlation results.

Variables	M	SD	1	2	3	4	5	6	7
1.age	21.02	1.93	-						
2.gender	.78	.41	-.026	1					
3.partner phubbing	2.75	.58	.090**	-.091**	1				
4.relationship satisfaction	3.75	.84	.018	-.124**	-.105**	1			
5.attachment anxiety	3.62	1.20	-.038	-.015	.182**	-.107**	1		
6.voice	6.25	1.55	.012	.035	-.042	.329**	.079*	1	
7.loyalty	4.30	1.58	.043	-.223**	.131**	.019	.420**	.158**	1

Table 2. The regression analysis of the moderated mediating model (Loyalty).

Dependent Variable	Independent Variable	R^2	F	β	*Bootstrap LLCI*	*Bootstrap ULCI*	t
Attachment Anxiety	Gender			.263(.092)	.082	.444	2.852**
	Age			-.042(.019)	-.079	-.004	-2.158*
	Partner Phubbing	.205	42.88***	.288(.065)	.161	.415	4.455***
	Loyalty			.322(.024)	.275	.370	13.268***
	Partner Phubbing× Loyalty			.0003(.031)	-.060	.991	.0114
Relationship Satisfaction	Gender			-.253(.072)	-.394	-.113	-3.544**
	Age			.008(.015)	-.022	.037	.523
	Partner Phubbing	.047	6.798***	-.157(.05)	-.256	-.058	-3.112**
	Attachment Anxiety			-.072(.027)	-.125	-.019	-2.684**
	Loyalty			.037(.021)	-.004	.078	1.752
	Attachment Anxiety × Loyalty			-.029(.012)	-.052	-.007	-2.538*

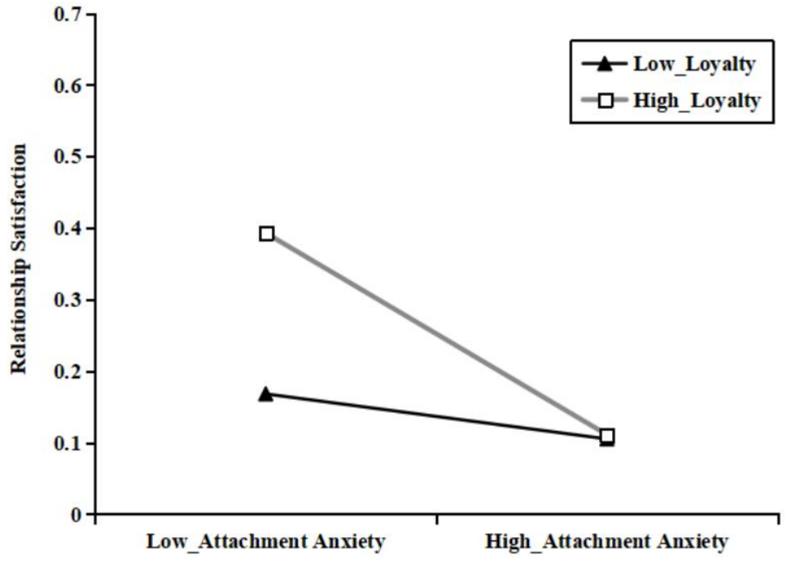

Figure 2. The association between attachment anxiety and relationship satisfaction for different level of loyalty.

Table 3. The conditional indirect effect analysis (Loyalty).

The Level of loyalty	Effect	BootSE	BootLLCI	BootULCI
M - SD	-.0076	.0121	-.0346	.0149
M	-.0208	.0103	-.0442	-.0041
M + SD	-.0341	.0134	-.0642	-.0111

Table 4. The regression analysis of the moderated mediating model (Voice).

Dependent Variable	independent Variable	R^2	F	β	*Bootstrap LLCI*	*Bootstrap ULCI*	t
Attachment Anxiety	Gender			-.002(.099)	-.196	.192	-.018
	Age			-.035(.021)	-.076	.007	-1.655
	Partner Phubbing	.045	7.758***	.399(.071)	.260	.538	5.632***
	Voice			.070(.026)	.018	.121	2.649**
	Partner Phubbing × Voice			.032(.038)	-.043	.107	.836
Relationship Satisfaction	Gender			-.302(.065)	-.431	-.174	-4.626***
	Age			.006(.014)	-.022	.033	.405
	Partner Phubbing	.159	6.798***	-.123(.047)	-.215	-.030	-2.590**
	Attachment Anxiety			-.084(.023)	-.1294	-.0393	-3.676***
	Voice			.172(.018)	.1372	.2076	9.6193***
	Attachment Anxiety × Voice			-.033(.013)	-.058	-.009	-2.653**

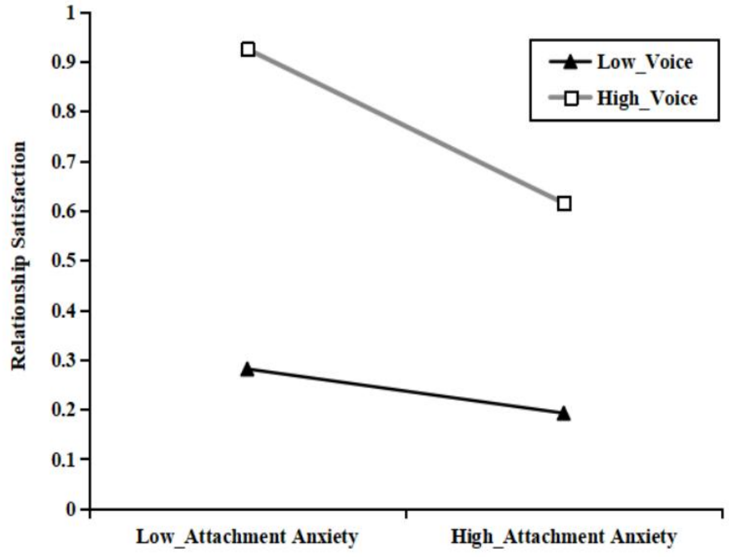

Figure 3. The association between attachment anxiety and relationship satisfaction for different level of voice.

Table 5. The conditional indirect effect analysis (Voice).

The Level of voice	Effect	BootSE	BootLLCI	BootULCI
M - SD	-.0114	.0140	-.0427	.0137
M	-.0337	.0123	-.0607	-.0129
M + SD	-.0611	.0228	-.1111	-.0214

Thank you for your attention !

Dr. Han Yichu

School of Teachers Education,
Huzhou University

May 20th, 2024
